# Surgery for Superior Sulcus Tumor, Partially Using a Robotic Approach, After Neoadjuvant Nivolumab With Platinum-Based Chemotherapy

**DOI:** 10.1016/j.atssr.2025.09.002

**Published:** 2025-10-03

**Authors:** Hitoshi Igai, Akinobu Ida, Kazuki Numajiri, Kazuhito Nii, Mitsuhiro Kamiyoshihara

**Affiliations:** 1 Department of General Thoracic Surgery, Japanese Red Cross Maebashi Hospital, Maebashi, Japan

## Abstract

Superior sulcus tumors have traditionally been treated with concurrent chemoradiotherapy followed by surgical resection, although radiation-induced fibrosis complicates surgery. Recent studies suggest that neoadjuvant nivolumab combined with platinum-based chemotherapy improves prognosis while limiting perioperative complications. We successfully performed surgical resection of a superior sulcus tumor by using a hybrid approach: an L-shaped incision for chest wall resection and a robotic-assisted technique for left upper lobectomy and lymphadenectomy. The patient achieved a major pathologic response with an uneventful recovery. This case highlights the potential of neoadjuvant immunochemotherapy and robotic surgery in optimizing outcomes for superior sulcus tumor management.

The gold standard treatment for superior sulcus tumors has been concurrent chemoradiotherapy followed by surgical resection because multiple studies have demonstrated excellent perioperative outcomes and favorable prognoses with this approach.^1^ However, surgical resection after radiotherapy remains technically challenging as a result of radiation-induced inflammation and fibrosis in the lung and surrounding tissues. These changes can compromise tissue integrity, thus making surgical manipulation more difficult and increasing the risk of complications.

Forde and colleagues^2^ reported that nivolumab combined with platinum-based chemotherapy, followed by surgical resection, resulted in superior prognoses, including improved event-free survival, higher pathologic complete response rates, and greater overall survival, compared with chemotherapy alone followed by surgical resection. Consequently, the number of patients with advanced non-small cell lung cancer who are undergoing this sequential treatment is increasing. Moreover, compared with preoperative chemoradiotherapy, which induces inflammation in the tumor and surrounding tissues, preoperative immunochemotherapy primarily targets the tumor itself. Therefore, in superior sulcus tumors, where major vessels are in close proximity, preoperative immunochemotherapy is expected to reduce the technical challenges associated with subsequent surgery.

Here we describe our successful surgical resection of a superior sulcus tumor in which we partially used a robotic approach after neoadjuvant nivolumab combined with platinum-based chemotherapy, and we report the details of the procedure.

## Technique

**Figure 1:**
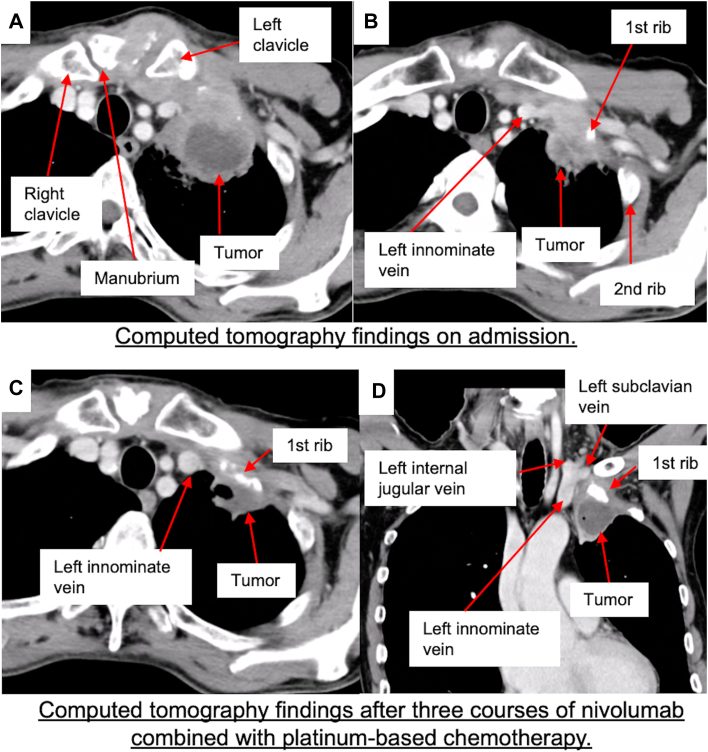
(A, B) Computed tomographic findings on admission. A 107-mm solid mass was observed in the left upper lobe that invaded the manubrium, the left clavicle (A), and the left first and second ribs (B), although no intrathoracic lymph node swelling was detected. (C, D) Computed tomographic findings after immunochemotherapy. The primary tumor showed remarkable shrinkage; however, invasion into the first rib persisted (C). Involvement of the great vessels was unclear (D).

A 67-year-old man with no significant medical history presented to our department with a 107-mm solid mass in the left upper lobe of the lung that invaded the manubrium, the left clavicle, and the first and second ribs, without evidence of intrathoracic lymph node enlargement, as revealed by computed tomography (CT) (Figures 1A, 1B). A CT-guided needle biopsy performed at our institution (Japanese Red Cross Maebashi Hospital, Maebashi, Japan) confirmed the diagnosis of squamous cell carcinoma. Immunohistochemical analysis of the biopsy specimen revealed a programmed death-ligand 1 tumor proportion score of 1%. Fluorodeoxyglucose (FDG) positron emission tomography (PET) demonstrated high FDG uptake in the primary tumor. On the basis of these findings, the clinical stage was determined to be cT4 N0 M0, stage IIIA. Surgical resection after neoadjuvant nivolumab combined with platinum-based chemotherapy was planned.

The patient initially underwent 3 cycles of neoadjuvant therapy with nivolumab in combination with carboplatin and paclitaxel. Follow-up CT revealed significant tumor shrinkage (Figures 1C, 1D). Additionally, FDG PET showed resolution of the previously high FDG uptake in the primary tumor. Consequently, the tumor was downstaged to ycT3 N0 M0, stage IIB, because its size decreased to 41 mm.

**Figure 2:**
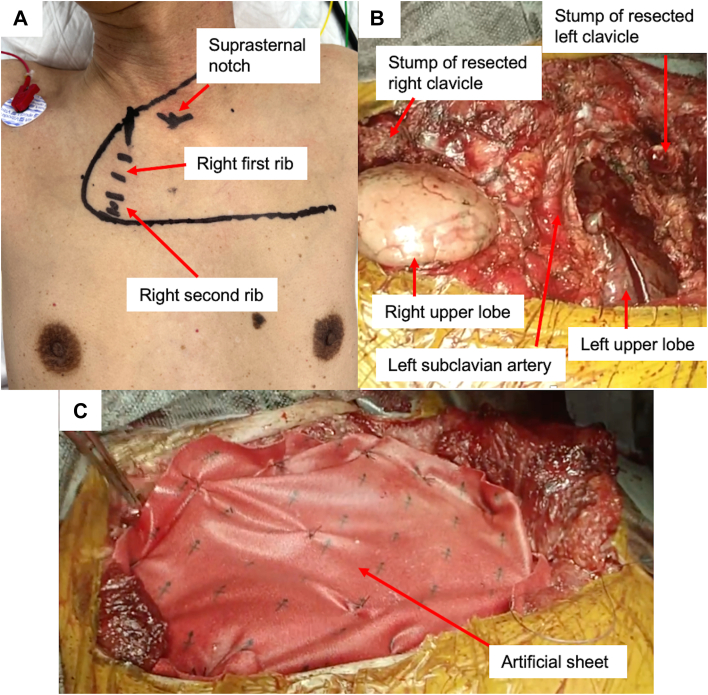
(A) L-shaped skin incision around the neck and manubrium. (B) Operative view after resection of the chest wall invaded by the tumor. (C) Defect reconstructed using an artificial sheet.

The Video demonstrates the surgical procedure and postoperative upper limb motion. The surgical procedure was initially performed with the patient in the supine position and using general anesthesia. Figure 2A shows the L-shaped skin incision made around the neck and manubrium. First, the right clavicle, as well as the first and second ribs, were transected with an adequate surgical margin after exposure of the chest wall. Subsequently, the sternum was horizontally divided with a saw at the third intercostal space after transection of the left second rib. The left clavicle and first rib were then transected with an adequate surgical margin. Because the tumor had invaded the left innominate, internal jugular, and subclavian veins, each vein was transected using a vascular stapler. Finally, the involved portion of the chest wall and the affected part of the left upper lobe were resected through a wide wedge resection (Figure 2B). The resulting defect was reconstructed using an artificial sheet (Figure 2C). The patient was then repositioned to the lateral decubitus position. The remaining left upper lobe was removed by a robotic-assisted approach with systemic radical lymphadenectomy (ND2a-2 lymphadenectomy), using 5 ports, including 1 port for an assistant surgeon.

**Figure 3:**
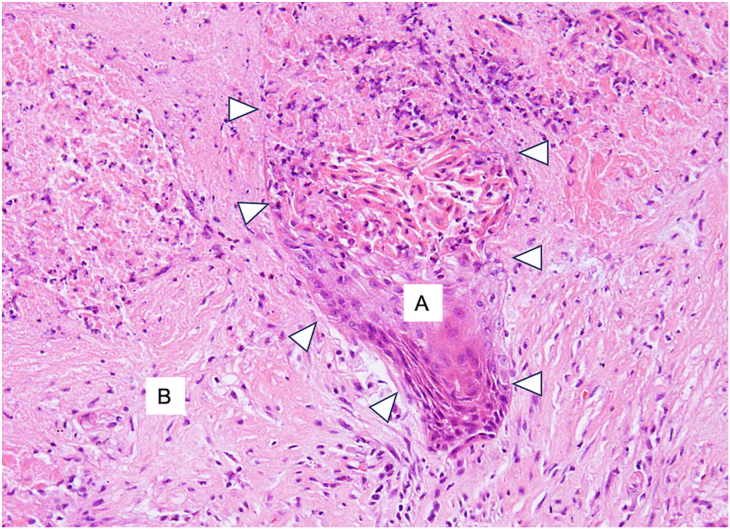
Histologically, a small number of viable atypical squamous epithelial cells was observed. A mixture of regions was identified: (A) areas with cellular atypia, suggesting the persistence of squamous cell carcinoma, and (B) areas where cellular atypia was less pronounced, indicating metaplastic squamous epithelium modified by treatment. Arrowheads indicate the border between these 2 regions. Hematoxylin and eosin stain; original magnification ×100.

The total operative time was 410 minutes. The chest tube was removed on postoperative day 1, and the patient was successfully discharged on postoperative day 10. There were no postoperative morbidities affecting either upper limb. The final pathology report confirmed ypT3 N0 M0, stage ⅡB squamous cell carcinoma, indicating a major pathologic response (Figure 3). The residual carcinoma was minimal, accounting for less than 10% of the tumor, and was located primarily in the wedge-resected lung specimen with direct invasion into the chest wall. No lymph node metastasis was identified.

## Comment

We successfully performed surgical resection of a superior sulcus tumor, by partially using a robotic approach after neoadjuvant nivolumab combined with platinum-based chemotherapy, despite the presence of inflammatory changes induced by therapy in certain areas. Moreover, the final pathology report revealed a major pathologic response, which may contribute to a favorable prognosis for the patient. However, not all tumors exhibit radiologic size reduction, and downstaging is not universally observed.

Although the Neoadjuvant Study of Nivolumab Plus Ipilimumab or Nivolumab Plus Chemotherapy vs Chemotherapy Alone in Early Stage Non-Small Cell Lung Cancer (CheckMate 816) trial by Forde and colleagues^2^ did not specifically include patients with superior sulcus tumors, it demonstrated the feasibility and efficacy of neoadjuvant chemoimmunotherapy in resectable, locally advanced non-small cell lung cancer. On the basis of this evidence, we adopted a similar neoadjuvant strategy for the present case, resulting in a favorable pathologic response and successful resection. In this patient, we observed that fibrosis was not extensive, and chest wall resection was relatively straightforward. However, to date, no studies have directly compared the extent of hilar or extrapulmonary fibrosis between neoadjuvant chemoimmunotherapy and conventional chemoradiotherapy. Therefore, the potential advantage of reduced fibrosis with chemoimmunotherapy remains speculative, and further investigation is warranted to clarify its impact on surgical difficulty and long-term outcomes.

Although the L-shaped skin incision provided an effective approach for resecting the invaded chest wall, it was insufficient for performing left upper lobectomy and lymphadenectomy. To address this issue, we applied a robotic approach, which required only 5 small ports (8-15 mm in diameter) on the lateral thorax, to complete the left upper lobectomy and lymphadenectomy after chest wall resection. Although an anterior thoracotomy at a lower intercostal space (eg, third or fourth) could possibly have allowed for lobectomy through the same incision, it would have necessitated wide rib spreading to obtain an adequate field, thereby increasing the surgical trauma. By contrast, the robotic approach enabled complete anatomic lobectomy and lymphadenectomy without the need for excessive rib spreading, thus reducing overall invasiveness and enhancing postoperative recovery. In a previous report, Kamel and colleagues^3^ demonstrated that a minimally invasive approach resulted in superior perioperative outcomes and long-term prognosis in patients with non-small cell lung cancer who underwent neoadjuvant chemotherapy followed by surgical resection, compared with open thoracotomy. This less invasive approach may have contributed to the patient’s favorable postoperative recovery.

The optimal timing of surgery after neoadjuvant chemoimmunotherapy remains unclear. Although many prospective clinical trials have adopted protocols scheduling surgery 4 to 6 weeks after the final dose, this interval is not yet evidence based.^2^^,^^4^ A prolonged delay may lead to excessive fibrosis, particularly in the tumor-invaded regions, that could potentially complicate complete resection. In support of earlier surgical intervention, Gu and colleagues^5^ retrospectively demonstrated that a shorter interval between neoadjuvant therapy and surgery was significantly associated with improved disease-free survival. These findings suggest that minimizing the interval while ensuring adequate patient recovery may help optimize both oncologic and technical outcomes.
